# Correction: Yin et al. Molecular Detection of *Babesia gibsoni* in Cats in China. *Animals* 2022, *12*, 3066

**DOI:** 10.3390/ani13010138

**Published:** 2022-12-29

**Authors:** Fangyuan Yin, Daoe Mu, Zhuojia Tian, Dong Li, Xiting Ma, Jinming Wang, Guiquan Guan, Hong Yin, Facai Li

**Affiliations:** 1 College of Veterinary Medicine, Southwest University, Chongqing 400715, China; 2 State Key Laboratory of Veterinary Etiological Biology, Lanzhou Veterinary Research Institute, Chinese Academy of Agricultural Science, Lanzhou 730046, China

## Title Correction

In the original publication [1], the corrected title should be “Molecular Detection of *Babesia gibsoni* in Cats in China”.

## Text Correction

In the Simple Summary section, the original sentence “…and the first case in China of *Babesia gibsoni* in cats was detected” should be replaced with “…and *Babesia gibsoni* was detected”.

In the Abstract section, the original sentence “This is the first report of *B. gibsoni* in cats in China” should be deleted.

In the Discussion section, a correction is needed for the first sentence in paragraph 1; the original sentence “…*B. vogeli* was found in three of 203 cats in China [20], …” should be replaced with “…*B. vogeli* was found in three of 203 cats in China [22], …”

In the Discussion section, a correction is needed for the last sentence in paragraph 1; the original sentence “For the presence of *B. gibsoni* in cat, there was just one reported in St Kitts [21], and *B. gibsoni*…” should be replaced with “For the presence of *B. gibsoni* in cats, there were related reports in China, Singapore and St Kitts [21,33]. *B. gibsoni*…”. 

In the Discussion section, a correction is needed for the last sentence in paragraph 2; the original sentence “This is the first report of molecular evidence for the existence of *B. gibsoni* in cats in China” should be deleted.

In the Conclusions section, the original sentence “*B. gibsoni* was found in a low proportion of asymptomatic cats in China for the first time…” should be replaced with “*B. gibsoni* was found in a low proportion of asymptomatic cats in China…”.

## Figure Correction

We added these four sequences (MN689645–MN689648) in Figure 4. 

The corrected Figure 4 appears below.

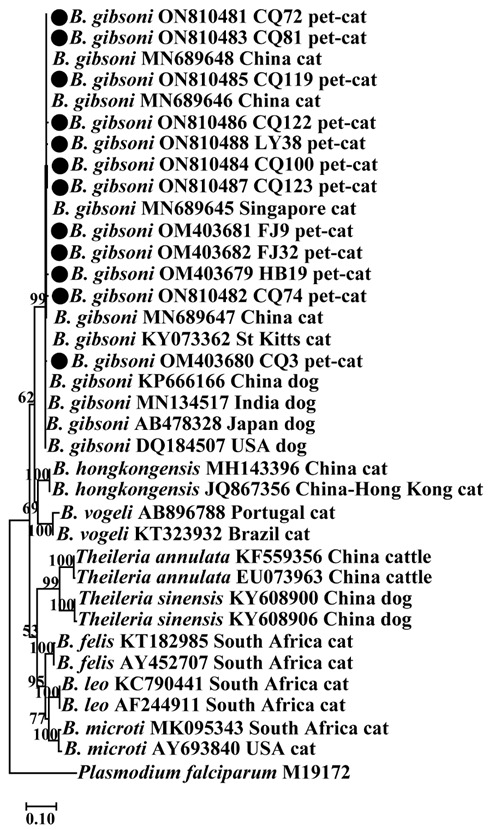



## References Correction

In the Discussion section, citation [33] is a new reference, as follow:

33. Colella, V.; Nguyen, V.L.; Tan, D.Y.; Lu, N.; Fang, F.; Yin, Z.; Wang, J.; Liu, X.; Chen, X.; Dong, J.; et al. Zoonotic vectorborne pathogens and ectoparasites of dogs and cats in eastern and southeast Asia. *Emerg. Infect. Dis.* **2020**, *26*, 1221–1233.

Original references order: 33–39.

Revised references order: 34–40.

With this correction, the order of some references has been adjusted accordingly.

The authors state that the scientific conclusions are unaffected. This correction was approved by the Academic Editor. The original publication has also been updated.
